# Impact of Cross-Tie Properties on the Modal Behavior of Cable Networks on Cable-Stayed Bridges

**DOI:** 10.1155/2015/989536

**Published:** 2015-06-17

**Authors:** Javaid Ahmad, Shaohong Cheng, Faouzi Ghrib

**Affiliations:** Department of Civil and Environmental Engineering, University of Windsor, 401 Sunset Avenue, Windsor, ON, Canada N9B 3P4

## Abstract

Dynamic behaviour of cable networks is highly dependent on the installation location, stiffness, and damping of cross-ties. Thus, these are the important design parameters for a cable network. While the effects of the former two on the network response have been investigated to some extent in the past, the impact of cross-tie damping has rarely been addressed. To comprehend our knowledge of mechanics associated with cable networks, in the current study, an analytical model of a cable network will be proposed by taking into account both cross-tie stiffness and damping. In addition, the damping property of main cables in the network will also be considered in the formulation. This would allow exploring not only the effectiveness of a cross-tie design on enhancing the in-plane stiffness of a constituted cable network, but also its energy dissipation capacity. The proposed analytical model will be applied to networks with different configurations. The influence of cross-tie stiffness and damping on the modal response of various types of networks will be investigated by using the corresponding undamped rigid cross-tie network as a reference base. Results will provide valuable information on the selection of cross-tie properties to achieve more effective cable vibration control.

## 1. Introduction

Stay cables are important load carrying structural elements on cable-stayed bridges. Due to high tension, friction between the composing wires or strands of stay cables is changed considerably which significantly reduces their structural damping [1–3]. This, along with their long and flexible feature, renders cables on cable-stayed bridges very vulnerable to various dynamic excitations. Over the past few decades, the length of stay cables has been increasing noticeably owing to the rapid growth of bridge span length. Therefore, using external dampers to suppress cable vibrations turns to be less effective because of the constraint on installation location. But rather, the cross-tie solution, which connects a vulnerable cable with its neighbouring ones using transverse cross-ties to form a cable network, becomes more popular in designing new cable-stayed bridges and rehabilitating the existing ones [4, 5].

Though the geometric form of a cable network appears to be relatively simple, its behaviour is highly sophisticated and is yet to be fully understood. The effectiveness of a cross-tie solution in improving stiffness and damping properties of a vulnerable cable greatly depends on the properties of cross-ties and the connected neighbouring cables. Up till now, much of the research effort was dedicated to investigate the enhancement on network in-plane stiffness resulting from the cross-tie solution [6–9]; only very few researchers explored how it would help to redistribute energy among different consisting cables [10] and affect the damping property of the network [11, 12]. While the undamped rigid cross-tie assumption was made in most of the cable network analytical models [6, 13], a few experimental and analytical studies were conducted to investigate the effect of cross-tie stiffness on the network behaviour. Scaled cable network models were tested by Yamaguchi and Nagahawatta [3] as well as Sun et al. [14]. It was found in both tests that the stiff type of cross-tie was more effective in improving network in-plane stiffness whereas the flexible one was more helpful in dissipating energy and increasing network damping. A study by Caracoglia and Jones [7] on a cable network on the Fred Hartman Bridge showed that if flexible cross-ties were selected instead of the rigid ones, the network fundamental frequency would decrease by 3%. On the contrary, Bosch and Park [15] pointed out in a numerical study that using oversized (too stiff) cross-tie would excite more local modes. This was confirmed by Ahmad and Cheng [8] who proposed an analytical model to investigate the impact of cross-tie stiffness on the dynamic response of a cable network. In the model, the main cables were assumed as taut cables and the flexible cross-tie was assumed to behave like a reversible tension/compression linear spring connector. It was observed that when a more flexible cross-tie was used, the local modes dominated by either the left or the right segments of a corresponding cable network using stiffer cross-tie would evolve into global modes. Giaccu and Caracoglia [16] addressed the nonlinear interaction between main cables and cross-ties with behaviour of the latter described by a generalized power-law stiffness model. They also reported that the equivalent nonlinear effects in network higher modes could be responsible for the transition from a local mode to a global one.

It is clear from the above literature review that connecting a vulnerable cable with its neighbouring ones through transverse cross-ties would alter its in-plane stiffness and damping property. The level of such alteration highly depends on cross-tie properties. When designing a cable network, the selection of cross-tie properties should be based on their combined impact on the network in-plane stiffness and damping. To properly assess the latter, the damping property of main cables as well as the stiffness and damping of cross-ties should be included in the analysis. The few available experimental studies (e.g., [3, 14]) only discussed how the first modal damping ratio of a vulnerable cable would be affected in a cable network, but such an impact on higher order modes was not addressed. Although a recent analytical study by the authors [12] examined this issue, the proposed model only considered damping in main cables, whereas the cross-tie was assumed to be undamped and rigid. Thus, the response between a vulnerable cable and its neighbouring ones was “communicated” directly without the influence of cross-tie properties. However, in reality, when transmitting responses between these two parties, cross-tie would behave like a “filter” which alters the transmitted response by its stiffness and damping properties. Therefore, to properly assess the effect of cross-tie solution on the dynamic response of a vulnerable cable and the formed cable network, it is imperative to describe the cross-tie behaviour more accurately.

In view of the above needs, the current paper aims at extending the analytical model proposed earlier by the authors [12] to include both the stiffness and damping properties of cross-tie in the formulation. The network system characteristic equation will be derived analytically and the modal characteristics of the cable network will be determined by solving the associated complex eigenvalue problem. The proposed model would allow evaluating the impact of cross-tie solution on the in-plane frequency and also the damping property of a vulnerable cable, not only for the fundamental mode, but for the higher order modes as well. The respective role of cross-tie stiffness and damping in affecting network dynamic behaviour will be examined. These would further enhance our understanding of cable network mechanics and provide valuable information to improve the current practice of cross-tie design.

## 2. Formulation of Analytical Model

**Figure 1:**
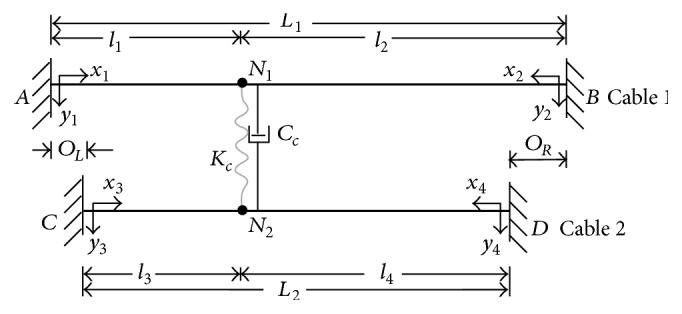
Schematic layout of a two-cable network with damped flexible cross-tie.

The proposed analytical model consists of two horizontally laid main cables connected by a transverse cross-tie, as shown schematically in Figure 1. The two main cables are assumed to be damped taut cables and are fixed at both ends. The length of the two cables is *L*_1_ and *L*_2_ (*L*_1_ ≥ *L*_2_), respectively. It is assumed that the longer cable (main cable 1) is vulnerable to dynamic excitations. In the following discussion, it will be referred to as the “target cable” whereas the shorter cable (main cable 2) will be referred to as the “neighbouring cable.” *O*_*L*_ and *O*_*R*_ denote, respectively, the left and the right horizontal offset of the neighbouring cable. In general, they are not equal to each other. The unit mass, tension, and structural damping ratio of the two main cables are denoted by *m*_*j*_, *H*_*j*_, and *ξ*_*j*_  (*j* = 1,2), respectively. The transverse cross-tie is assumed to be located at *l*_1_  (*l*_1_ < *l*_2_) from the left end of the target cable. Its axial stiffness property is represented by a linear spring connector with an associated stiffness constant of *K*_*c*_, where the subscript “*c*” refers to “cross-tie.” The structural damping, which comes from various types of energy dissipation mechanisms, is usually represented by a highly idealized form in structural analysis. The equivalent linear viscous damping model proposed by Rayleigh [17] assumes the damping force to be linearly proportional to the motion velocity. This model is commonly adopted due to the simplicity of its form and the amenability in deriving analytical solution. Therefore, in the current model, damping property of the two main cables and that of the cross-tie are all assumed to be the linear viscous type and uniformly distributed along the member length. The corresponding damping coefficients are denoted *C*_*j*_  (*j* = 1,2) for the *j*th main cable and *C*_*c*_ for the cross-tie. The additional tension in the main cables caused by vibration is neglected in the proposed model.

When the cable network in Figure 1 is excited to vibrate within its plane, all four main cable segments oscillate in the transverse direction whereas the cross-tie moves along its longitudinal direction. The motion of each main cable segment can be described by the equation of motion of a taut cable subjected to in-plane damped free vibration; that is,

(1) $$\begin{array}{c} H\frac{\partial^{2}v}{\partial x^{2}}=m\frac{\partial^{2}v}{\partial t^{2}}+C\frac{\partial v}{\partial t} \end{array}$$

or

(2) $$\begin{array}{c} H\frac{\partial^{2}v}{\partial x^{2}}=m\frac{\partial^{2}v}{\partial t^{2}}+2m\xi \omega_{o}\frac{\partial v}{\partial t}, \end{array}$$

where *v*, *H*, and *m* are, respectively, the transverse displacement, the tension, and the unit mass of the taut cable, *C* = 2*mξω*_*o*_ is the cable damping coefficient per unit length, *ξ* is the cable structural damping ratio, and *ω*_*o*_ is the undamped circular frequency of the taut cable. Separating the temporal and spatial variables contained in the cable transverse displacement *v*(*x*, *t*) using the Bernoulli-Fourier method, it can be expressed as $v\left(x,t\right)=\overset{-}{v}\left(x\right)e^{i\omega t}$, where $\overset{-}{v}\left(x\right)$ is the shape function and *ω* is the complex circular frequency of cable vibration. Substituting this expression into (2), it becomes

(3) $$\begin{array}{c} {\overset{-}{v}}^{\prime \prime }\left(x\right)+\alpha^{2}\overset{-}{v}\left(x\right)=0 \end{array}$$

and its general solution would be

(4) $$\begin{array}{c} \overset{-}{v}\left(x\right)=A\cos\left(\alpha x\right)+B\sin\left(\alpha x\right), \end{array}$$

where *A* and *B* are constants determined from the boundary conditions and *α* is a complex wave number of the form

(5) $$\begin{array}{c} \alpha =\sqrt{\frac{m\omega^{2}-i\cdot 2m\xi \omega \omega_{o}}{H}}. \end{array}$$

Since all four main cable segments in Figure 1 have one end fixed, that is, $\overset{-}{v}\left(0\right)=0$, constant *A* in (4) would be zero. Therefore, their transverse motion shape functions can be reduced to

(6a) $$\begin{array}{c} {\overset{-}{v}}_{2j-1}\left(x_{2j-1}\right)=B_{2j-1}\sin\left(\alpha_{j}x_{2j-1}\right)\;j=1,2, \end{array}$$

(6b) $$\begin{array}{c} {\overset{-}{v}}_{2j}\left(x_{2j}\right)=B_{2j}\sin\left(\alpha_{2j}x_{2j}\right)\;j=1,2, \end{array}$$

where ${\overset{-}{v}}_{2j-1}$ and ${\overset{-}{v}}_{2j}$ represent, respectively, the shape function of the left and the right segments of the *j*th main cable (*j* = 1,2) and *B*_2*j*−1_ and *B*_2*j*_ are the corresponding shape function constants.

The mass of the cross-tie is not considered in the proposed model since it is usually very small compared to that of the main cables. The behaviour of the damped flexible cross-tie is described by a linear tension/compression reversal spring connector in parallel with a linear viscous damper. When the cross-tie oscillates along its axial direction, the force developed in it can be expressed as

(7) $$\begin{array}{c} {F_{c}\left(t\right)=K}_{c}u\left(t\right)+C_{c}\frac{\partial u}{\partial t}, \end{array}$$

where *u*(*t*) is the change in cross-tie length; that is,

(8) $$\begin{array}{c} u\left(t\right)=v_{1}\left(l_{1},t\right)-v_{3}\left(l_{3},t\right)=\left[{\overset{-}{v}}_{1}\left(l_{1}\right)-{\overset{-}{v}}_{3}\left(l_{3}\right)\right]e^{i\omega t}. \end{array}$$

At point *N*_1_ where the cross-tie connects with the target cable (Figure 1), the equilibrium requires the force exerted by the cross-tie on the target cable to be equal to the transverse force in the left and the right segments of the target cable induced by its tension; that is,

(9) $$\begin{array}{c} \left[\left({\left.\frac{\partial {\overset{-}{v}}_{1}}{\partial x_{1}}\right|}_{x_{1}=l_{1}}+{\left.\frac{\partial {\overset{-}{v}}_{2}}{\partial x_{2}}\right|}_{x_{2}=l_{2}}\right)H_{1}\right]e^{i\omega t}=-F_{c}\left(t\right). \end{array}$$

Plug (7) and (8) into (9); it gives

(10) α1H1B1cos⁡α1l1+B2cos⁡α1l2 =B3sinα2l3−B1sinα1l1Kc+iωCc.

Moreover, longitudinal equilibrium of the isolated cross-tie should satisfy

(11) ∂v−1∂x1x1=l1+∂v−2∂x2x2=l2H1  +∂v−3∂x3x3=l3+∂v−4∂x4x4=l4H2=0.

Substitute (6a) and (6b) into (11); the following equation is obtained:

(12) α1H1B1cos⁡α1l1+B2cos⁡α1l2  +α2H2B3cos⁡α2l3+B4cos⁡α2l4=0.

The transverse displacement compatibility between the left and the right main cable segments at nodes *N*_1_ and *N*_2_ gives

(13) $$\begin{array}{c} {\overset{-}{v}}_{2j-1}\left(l_{2j-1}\right)={\overset{-}{v}}_{2j}\left(l_{2j}\right)\;j=1,2 \end{array}$$

which, by considering (6a) and (6b), yields

(14a) $$\begin{array}{c} B_{1}\sin\left(\alpha_{1}l_{1}\right)-B_{2}\sin\left(\alpha_{1}l_{2}\right)=0, \end{array}$$

(14b) $$\begin{array}{c} B_{3}\sin\left(\alpha_{2}l_{3}\right)-B_{4}\sin\left(\alpha_{2}l_{4}\right)=0. \end{array}$$

Combining (10), (12), (14a), and (14b) and expressing in a matrix form, we get

(15) $$\begin{array}{c} \left[S\right]\left\{X\right\}=\left\{0\right\}, \end{array}$$

where

(16) $$\begin{array}{c} \left[S\right]=\left[\begin{array}{cccc} \sin\left(\emptyset_{1}\right) & -\sin\left(\emptyset_{2}\right) & 0 & 0\\ 0 & 0 & \sin\left(\emptyset_{3}\right) & -\sin\left(\emptyset_{4}\right)\\ \psi R_{1}\cos\left(\emptyset_{1}\right)+\sin\left(\emptyset_{1}\right) & \psi R_{1}\cos\left(\emptyset_{2}\right) & -\sin\left(\emptyset_{3}\right) & 0\\ \gamma_{1}\cos\left(\emptyset_{1}\right) & \gamma_{1}\cos\left(\emptyset_{2}\right) & \gamma_{2}\cos\left(\emptyset_{3}\right) & \gamma_{2}\cos\left(\emptyset_{4}\right) \end{array}\right] \end{array}$$

is the coefficient matrix, $\{X\}={\left.\left[\begin{array}{cccc} B_{1} & B_{2} & B_{3} & B_{4} \end{array}\right.\right]}^{T}$ is a vector containing all four unknown shape function constants, and {0} is the null vector. In the coefficient matrix [*S*], *∅*_2*j*−1_ = *R*_*j*_ *ε*_2*j*−1_ and *∅*_2*j*_ = *R*_*j*_ *ε*_2*j*_ apply, respectively, to the left and the right segment of the *j*th main cable (*j* = 1,2), *R*_*j*_ = *α*_*j*_ *L*_*j*_ is a complex parameter, *α*_*j*_ is the complex wave number defined in (5), *ε*_2*j*−1_ = *l*_2*j*−1_/*L*_*j*_ and *ε*_2*j*_ = *l*_2*j*_/*L*_*j*_ are the segment ratio parameters for the left and the right cable segments of the *j*th main cable (*j* = 1,2), and *γ*_*j*_ is the mass-tension ratio parameter of the *j*th cable which is defined by

(17) $$\begin{array}{c} \gamma_{j}=\frac{H_{j}R_{j}/L_{j}}{H_{1}R_{1}/L_{1}}; \end{array}$$

*ψ* is the nondimensional complex cross-tie parameter having the form of

(18) $$\begin{array}{c} \psi =\frac{H_{1}}{L_{1}\left[K_{c}+i\omega C_{c}\right]}. \end{array}$$

Define Ω = *πf*/*f*_1_ as the nondimensional complex frequency of the cable network and *η*_*j*_ = *f*_1_/*f*_*j*_ as the frequency ratio of the *j*th (*j* = 1,2) main cable, where *f* and *f*_*i*_ are, respectively, the complex frequency of the cable network and the undamped fundamental frequency of the *j*th (*j* = 1,2) main cable; the complex parameter *R*_*j*_ can be rewritten as

(19) $$\begin{array}{c} R_{j}=\sqrt{\left[{\left(Ω\eta_{j}\right)}^{2}-i\cdot 2{\pi \xi }_{j}Ω\eta_{j}\right]}\;j=1,2, \end{array}$$

where *ξ*_*j*_  (*j* = 1,2) is the structural damping ratio of the *j*th cable.

To find the nontrivial solution to (15), the determinant of the coefficient matrix [*S*] should be set to zero. This leads to the characteristic equation of the two-cable network shown in Figure 1, which consists of two horizontally laid damped taut main cables interconnected by a transverse damped flexible cross-tie; that is,

(20) γ1sinR1sin∅3sin∅4  +γ2sinR2sin∅1sin∅2  +ψR1γ1γ2sinR1sinR2=0.

If we neglect the damping in the two main cables and the cross-tie, the three complex parameters *R*_*j*_, *γ*_*j*_  (*j* = 1,2), and *ψ* in (17) to (19) would reduce to *R*_*j*_ = Ω*η*_*j*_, $\gamma_{j}=\sqrt{H_{j}m_{j}/H_{1}m_{1}}$, and *ψ*_*o*_ = *H*_1_/*L*_1_ *K*_*c*_. Therefore, (15) would be the same as the system characteristic equation of an undamped two-cable network connected through an undamped flexible cross-tie derived earlier by the authors [8], and *ψ*_*o*_ is the cross-tie stiffness parameter defined in [8].

## 3. Case Studies

In this section, the proposed analytical model will be validated by finite element simulations and applied to study dynamic behaviour of cable networks having different layouts. By using the rigid cross-tie case as a reference base, the impact of cross-tie stiffness and damping on network modal responses will be evaluated.

A finite element model of the cable network shown in Figure 1 will be developed using the commercial finite element analysis software Abaqus 6.10. The behaviour of the main cables will be simulated by the two-node linear B21 beam element, whereas the flexible damped cross-tie will be modeled by the CONN2D2 connector element. The Rayleigh viscous damping model will be applied to simulate the structural damping in the main cables and cross-tie.

### 3.1. Case 1: Twin-Cable Network

In a twin-cable network, two main cables of the same geometric and physical properties are arranged in parallel with each other and interconnected by a transverse cross-tie. This type of cable network is not common in practice. However, due to its unique characteristics, seeking analytical solution of network modal response would be possible. Thus, studying dynamic behaviour of this special type of cable network has the merit of deepening our understanding of phenomena associated with cable networks.

Since the two main cables in a twin-cable network have the same length, unit mass, tension, and damping, it gives *∅*_1_ = *∅*_3_, *∅*_2_ = *∅*_4_, *R*_1_ = *R*_2_, and *γ*_1_ = *γ*_2_ = 1. By inserting these conditions into (20), the system characteristic equation can be reduced to

(21) $$\begin{array}{c} 2\sin\left(R_{1}\right)\sin\left(\emptyset_{1}\right)\sin\left(\emptyset_{2}\right)+\psi R_{1}\sin^{2}\left(R_{1}\right)=0 \end{array}$$

or

(22) $$\begin{array}{c} \sin\left(R_{1}\right)\left[2\sin\left(\emptyset_{1}\right)\sin\left(\emptyset_{2}\right)+\psi R_{1}\sin\left(R_{1}\right)\right]=0. \end{array}$$

Three sets of roots can be determined from (22). The first set, yielded from sin(*R*_1_) = 0, describes network global modes and is independent of cross-tie properties. Since the damping of main cables and cross-tie is considered in the current study, the network complex frequency can be expressed as

(23) $$\begin{array}{c} Ω={Ω}_{\text{re}}+i\cdot {Ω}_{\text{im}}, \end{array}$$

where ${Ω}_{\text{re}}={Ω}_{o}\sqrt{1-\xi_{\text{eq}}^{2}}$ and Ω_im_ = Ω_*o*_ *ξ*_eq_ are, respectively, the real and the imaginary parts of Ω, Ω_*o*_ is the nondimensional undamped network frequency, and *ξ*_eq_ is the equivalent damping ratio of the system. The real part of the complex frequency describes network vibration frequency whereas the imaginary part gives the system energy dissipation capacity. Substitute (23) into sin(*R*_1_) = 0; we obtain ${Ω}_{\text{re}}=n\pi \sqrt{1-{(\xi /n)}^{2}}$ and Ω_im_ = *πξ*, where *n* and *ξ* are, respectively, the mode number and damping ratio of an isolated single main cable. The nondimensional modal frequency Ω_0_ and modal damping ratio *ξ*_eq_ of the corresponding network global mode can thus be computed from

(24a) $$\begin{array}{c} {Ω}_{0}=\sqrt{{Ω}_{\text{re}}^{2}+{Ω}_{\text{im}}^{2}}=n\pi \;n=1,2,3,\ldots , \end{array}$$

(24b) $$\begin{array}{c} \xi_{\text{eq}}=\frac{{Ω}_{\text{im}}}{\sqrt{{Ω}_{\text{re}}^{2}+{Ω}_{\text{im}}^{2}}}=\frac{\xi }{n}\;n=1,2,3,\ldots . \end{array}$$

This set of network modal property is exactly the same as those of an isolated single cable, which suggests that, in this kind of global modes, the two main cables would oscillate in-phase with the same shape and the modal properties are not affected by the presence of cross-tie.

The other two sets of roots can be obtained by setting the summation of the two terms in the square bracket of (22) to zero; that is,

(25) $$\begin{array}{c} 2\sin\left(\emptyset_{1}\right)\sin\left(\emptyset_{2}\right)+\psi R_{1}\sin\left(R_{1}\right)=0. \end{array}$$

It is important to note that should a rigid cross-tie be used in a twin-cable network, that is, *ψ* = 0, the second term in (25) would vanish. Therefore, the two remaining sets of roots of (22) can be directly obtained from sin(*∅*_1_) = 0 and sin(*∅*_2_) = 0, which represent, respectively, the network local modes dominated by its left segments (LS) or right segments (RS). However, if the cross-tie has certain flexibility and damping, the second term in (25) would reflect the effect of cross-tie properties (stiffness, damping, and position) on the network local modes. Not only would their modal frequencies and damping be “modified,” but also their mode shapes would evolve from that dominated by the oscillations of either the network left or right segments to the out-of-phase global modes. This agrees with the earlier findings by the authors [8] when studying two identical taut main cables interconnected by an undamped flexible cross-tie.

When installing a cross-tie, it can be placed either at the nodal point of a specific main cable mode or off the nodal point. Noticing *R*_1_ = *∅*_1_/*ε*_1_ = *∅*_2_/*ε*_2_, (25) can also be expressed as

(26) $$\begin{array}{c} 2\sin\left(\emptyset_{1}\right)\sin\left(\emptyset_{2}\right)+\psi R_{1}\sin\left[\frac{1}{m}\frac{\emptyset_{1}}{\varepsilon_{1}}+\left(1-\frac{1}{m}\right)\frac{\emptyset_{2}}{\varepsilon_{2}}\right]=0, \end{array}$$

where *m* is a positive integer or fraction which would satisfy *mε*_1_ = 1 and thus (1 − 1/*m*) = *ε*_2_ when cross-tie happens to be placed at the nodal point of a particular main cable mode. Therefore, (26) can be further simplified as

(27) sin∅12sin∅2+ψR1cos⁡∅2+cos⁡∅1sin(∅2)sin∅1  =0.

In (27), the condition of sin(*∅*_1_) = 0 describes the counterpart of LS modes in a rigid cross-tie network, the modal frequency and damping ratio of which are

(28a) $$\begin{array}{c} {Ω}_{0}=\frac{n\pi }{\varepsilon }\;n=1,2,3,\ldots , \end{array}$$

(28b) $$\begin{array}{c} \xi_{\text{eq}}=\frac{\varepsilon \xi }{n}\;n=1,2,3,\ldots . \end{array}$$

This implies that if a damped flexible cross-tie is placed at the nodal point of a particular main cable mode, although the change in cross-tie properties would lead to evolution of mode shapes into out-of-phase globe modes, the modal frequency and damping of these modes will not be affected.

The roots obtained by setting the term enclosed by the curly bracket in (27) to zero reflect how modal properties of local RS modes in a rigid cross-tie case would be influenced by the flexibility and damping of a cross-tie. They would not only contribute to reduce modal frequency and increase modal damping, but also excite more sizable oscillations of the left segments. Therefore, a network local RS mode would evolve into a global one should a rigid cross-tie be replaced by a damped flexible one. The same mode evolution phenomenon was reported earlier [8] for a twin-cable network with an undamped flexible cross-tie located at the quarter span of the main cables, which is the nodal point of the second antisymmetric main cable mode.

From the above discussion, it is clear that, in a twin-cable network, the in-phase global modes are independent of the cross-tie position, stiffness, and damping. However, modal properties of local RS modes in the rigid cross-tie case would be “modified” by the cross-tie stiffness and damping and evolve into global modes. The impact of cross-tie stiffness and damping on the modal properties of local LS modes depends on the cross-tie installation location. If the cross-tie is located at the main cable nodal point, the frequency and damping of the LS modes would be independent of cross-tie stiffness and damping. However, if the cross-tie is not placed at the nodal point, the presence of cross-tie stiffness and damping would alter modal frequency and damping of the LS modes. In both cases, such a change in cross-tie properties would render a local LS mode evolving to an out-of-phase global mode.

#### 3.1.1. Numerical Example

To validate the proposed cable network analytical model and further discuss the modal characteristics associated with twin-cable networks, a numerical example is presented. Both main cables are assumed to have a length of 72 m, a unit mass of 50 kg/m, a tension of 2200 kN, and a structural damping ratio of 0.5%. The stiffness coefficient of cross-tie is assumed to be *K*_*c*_ = 30.54 kN/m, and its damping coefficient is *C*_*c*_ = 1.0 kN·s/m. Two cross-tie installation locations of *ε* = 1/3 and *ε* = 2/5 are considered in the example.

**Figure 2:**
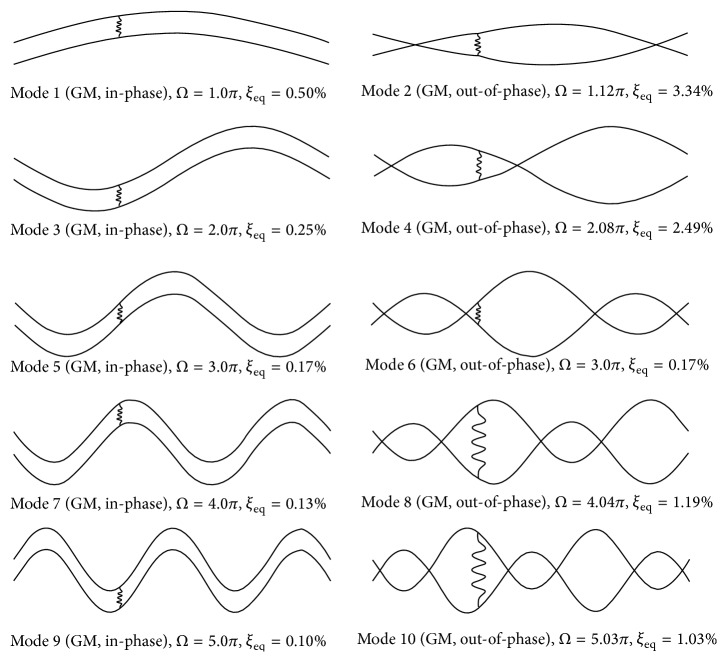
First ten modes of a twin-cable network with a damped flexible cross-tie (*K*_*c*_ = 30.54 kN/m, *C*_*c*_ = 1.0 kN·s/m) at *ε* = 1/3.

*(a) Cross-Tie Installed at ε* = 1/3. In this case, a flexible damped cross-tie is placed at the one-third span of the two main cables, which happens to be the nodal point of the 3rd mode of an isolated single main cable. The modal properties of the first ten network modes obtained from the proposed analytical model and finite element simulation are listed in Table 1, and the mode shapes are depicted in Figure 2. The two sets of results are found to agree well. Given also in the same table are the modal properties of the corresponding rigid cross-tie twin-cable network. It can be seen from the table that for all five in-phase global modes, that is, Modes 1, 3, 5, 7, and 9, their modal frequencies, modal damping ratios, and mode shapes not only are independent of the cross-tie flexibility and damping but also are not affected by the presence of cross-tie. The properties of these modes remain the same as those of an isolated single cable. Since the cross-tie is placed at the main cable one-third span, the modal frequency and damping of the first local LS mode, Mode 6, remain the same when the rigid cross-tie is replaced by a damped flexible one, but the mode shape evolves to an out-of-phase global mode. Moreover, this particular cross-tie position also renders the modal frequency and modal damping ratio of Mode 6 to be the same as those of Mode 5, which is the 3rd in-phase network global mode. In the case of Modes 2, 4, 8, and 10, which are pure local RS modes in the rigid cross-tie case, the adoption of a flexible damped cross-tie is found to not only considerably affect their modal frequencies and damping ratios, but also alter their mode shapes. For example, in the case of Mode 2, such a change in the cross-tie properties would excite the left segments of the network so that the mode shape becomes an out-of-phase global mode. The modal frequency is reduced by 25% from 2.18 Hz to 1.63 Hz, whereas the modal damping ratio increases substantially from 0.33% to 3.34% by roughly ten times. One possible reason for such a drastic increment in the network modal damping ratio could be the relatively high damping coefficient (*C*_*c*_ = 1.0 kN·s/m) and relatively low stiffness coefficient (*K*_*c*_ = 30.54 kN/m) assumed in the example. Besides, it is also important to note that since linear viscous type of damping model is used for the cross-tie, the energy dissipation due to damped cross-tie would only occur when the two ends of the cross-tie oscillate at different velocities. Therefore, in the case of network in-phase global modes (Modes 1, 3, 5, 7, and 9) of which the twin cables vibrate with the same shape, the oscillating velocities at the cross-tie two ends are the same so that a flexible damped cross-tie is not capable of dissipating energy. Thus, all the network in-phase global modes have the same modal damping ratio as that of an isolated single cable vibrating in the same mode. On the other hand, when a network out-of-phase global mode is excited, velocities at the two ends of the cross-tie are equal but opposite in direction, so the flexible damped cross-tie would manifest the maximum possible energy dissipation capacity. Similar pattern, that is, decrease in modal frequency and significant increase in modal damping, can also be found in higher order out-of-phase global modes (Modes 4, 8, and 10).

**Figure 3:**
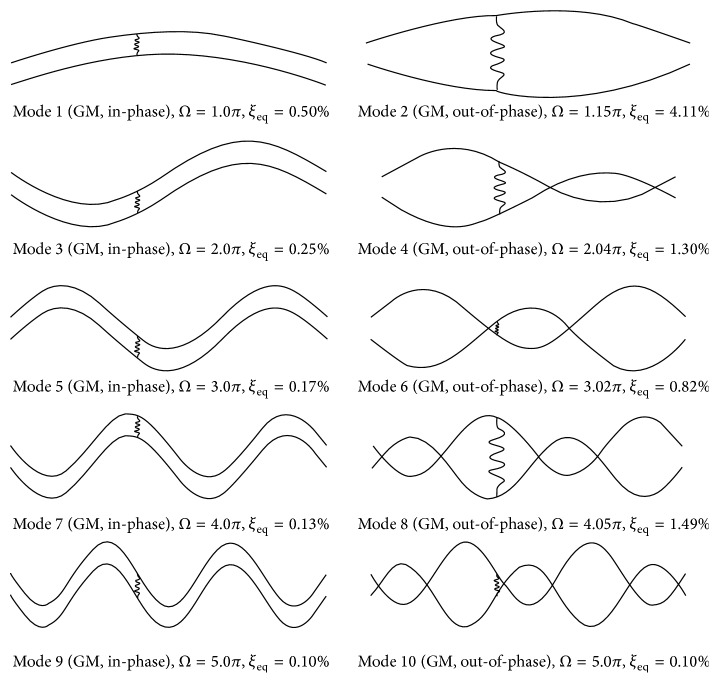
First ten modes of a twin-cable network with a damped flexible cross-tie (*K*_*c*_ = 30.54 kN/m, *C*_*c*_ = 1.0 kN·s/m) at *ε* = 2/5.

*(b) Cross-Tie Installed at ε* = 2/5. Modal analysis of the same twin-cable network is conducted by relocating the cross-tie position to *ε* = 2/5.  Table 2 summarizes the modal properties of the first ten network modes, and the mode shapes are portrayed in Figure 3. A good agreement between the modal results determined by the proposed analytical model and finite element simulations can be clearly observed from Table 2. For the convenience of comparison, the modal properties of the corresponding rigid cross-tie network are also listed in Table 2. Results show that, similar to the previous case of *ε* = 1/3, the modal characteristics (modal frequency, modal damping ratio, and mode shape) of the in-phase global modes, that is, Modes 1, 3, 5, 7, and 9, are not affected by the presence of cross-tie. They remain the same as the flexibility and damping of the cross-tie change. When using a rigid cross-tie network as a reference base, by increasing cross-tie flexibility and damping, modal frequency of local RS modes decreases whereas modal damping ratio increases. In addition, their mode shapes evolve to out-of-phase global modes. Take Mode 2 as an example, when replacing the rigid cross-tie by a flexible damped one with *K*_*c*_ = 30.54 kN/m and *C*_*c*_ = 1.0 kN·s/m, its frequency drops from 2.43 Hz to 1.68 Hz by 31%, but the associated modal damping ratio increases approximately 12.5 times from 0.33% to 4.11%, and the oscillation extends from the right segments to the entire network. The same phenomenon can be observed in Mode 6 and Mode 8. In the case of Mode 4 and Mode 10, both of which are local LS modes in the rigid cross-tie network, a cross-tie position of *ε* = 2/5 is off the nodal point of the 1st antisymmetric mode of an isolated cable in Mode 4, but it happens to be the nodal point of the 3rd symmetric mode of an isolated cable in Mode 10. Thus, for Mode 4, the change in cross-tie properties would not only cause evolution of its mode shape into an out-of-phase global mode, but also alter its modal frequency and damping ratio, whereas for Mode 10, its frequency and damping ratio remain the same as those of the rigid cross-tie case, although the mode evolution phenomenon occurs.

### 3.2. Case 2: Symmetric DMT Cable Network

In majority of cable networks on real cable-stayed bridges, the consisting main cables have different length, unit mass, and tension, which results in different mass-tension (DMT) ratio. Besides, since the spacing between cables is typically closer on the pylon side than on the deck side, the geometric layout of a real cable network is generally asymmetric. However, it is understood from earlier studies [8, 12] that if rigid or flexible undamped cross-ties are used in a symmetric cable network, pure local modes dominated by oscillations of individual main cables could form. Therefore, before analyzing a more realistic asymmetric DMT cable network, the impact of using flexible damped cross-ties on the modal response of a DMT cable network having symmetric layout is studied first.

When the cable network in Figure 1 has a symmetric layout, the left and the right offsets of main cable 2 are the same; that is, *O*_*L*_ = *O*_*R*_, and the flexible damped cross-tie locates at the mid-span of the two main cables. Therefore, the segment parameters would satisfy *∅*_1_ = *∅*_2_ = *R*_1_/2 and *∅*_3_ = *∅*_4_ = *R*_2_/2. Substitute these relations into (20); the system characteristic equation of a symmetric DMT two-cable network can be expressed as

(29) sinR12sinR22cos⁡R12sinR22hhhhhhhhhhhhhhhh+γ2sinR12cos⁡R22hhhhhhhhhhhhhhhhR12+2ψR1γ2sin⁡R1=0.

By setting each of the three terms on the left side of (29) to zero, that is, the two sine terms and the one enclosed by the square bracket, three sets of roots can be determined. The first two sets, yielded, respectively, from sin(*R*_1_/2) = 0 and sin(*R*_2_/2) = 0, describe the local modes dominated by the target or the neighbouring cable. They are as follows:

 Local modes of the target cable:
  (30a) $$\begin{array}{c} {Ω}_{o}=2n\pi \;n=1,2,3,\ldots , \end{array}$$
  (30b) $$\begin{array}{c} \xi_{\text{eq}}=\frac{\xi_{1}}{\left(2n\right)}\;n=1,2,3,\ldots . \end{array}$$
 Local modes of the neighboring cable:
  (31a) $$\begin{array}{c} {Ω}_{o}=\frac{2n\pi }{\eta_{2}}\;n=1,2,3,\ldots , \end{array}$$
  (31b) $$\begin{array}{c} \xi_{\text{eq}}=\frac{\xi_{2}}{\left(2n\right)}\;n=1,2,3,\ldots . \end{array}$$

First of all, it is interesting to note that the form of (30a), (30b) and (31a), (31b) implies that these two types of network local modes have the same modal properties as the respective isolated single cable antisymmetric modes. Secondly, the same two types of network local modes, with exactly the same modal frequencies and modal damping ratios, were identified earlier [12] when analyzing modal behaviour of a symmetric DMT two-cable network using rigid cross-tie. Clearly, since the cross-tie is placed at the nodal point of the main cables, the network local modes dominated by an individual main cable would not be affected by the stiffness and damping of the cross-tie.

The third set of roots, describing the modal properties of network global modes, can be found by setting the term in the square bracket of (29) to zero. While the first two terms inside the square bracket show the interaction between the two main cables and the coupling in their motions, the third term reflects the role of cross-tie properties in “modifying” the frequency and damping of network global modes. Should a rigid cross-tie be used, this term would vanish, suggesting that the properties of network global modes would only be affected by the main cable properties. This set of solution can be determined by separating the real and imaginary terms and using the same procedure explained in Section 3.1 or [12].

#### 3.2.1. Numerical Example

Consider a symmetric DMT cable network with the following properties:

 Main cable 1:
  (32) $$\begin{array}{c} L_{1}=72\;\text{m},\;\;H_{1}=2200\;\text{kN},\;\;m_{1}=50\;\text{kg}/\text{m},\\ \xi_{1}=0.5\%. \end{array}$$
 Main cable 2:
  (33) $$\begin{array}{c} L_{2}=60\;\text{m},\;\;H_{2}=2400\;\text{kN},\;\;m_{2}=42\;\text{kg}/\text{m},\\ \xi_{2}=0.8\%. \end{array}$$
 Cross-tie:
  (34) $$\begin{array}{c} \varepsilon =\frac{1}{2},\;\;K_{c}=30.54\;\text{kN}/\text{m},\;\;C_{c}=1.0\;\text{kN}\cdot \text{s}/\text{m}. \end{array}$$

**Figure 4:**
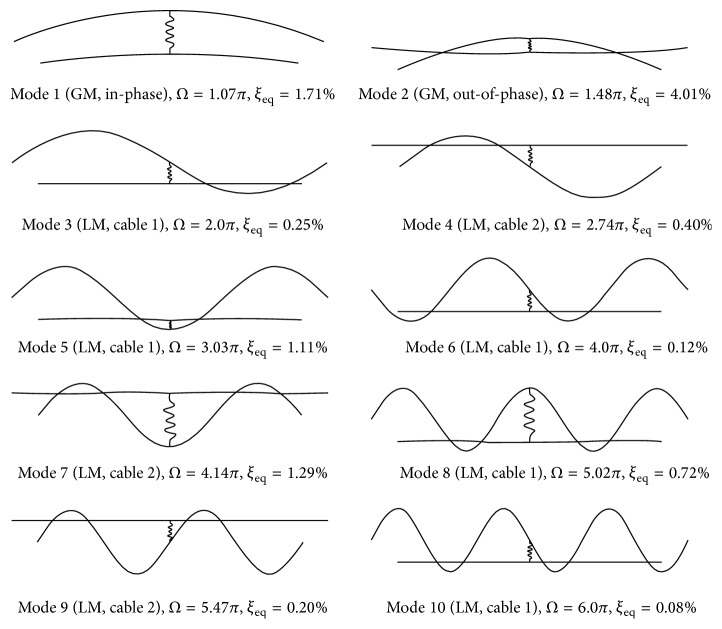
First ten modes of a symmetric DMT two-cable network with a damped flexible cross-tie (*K*_*c*_ = 30.54 kN/m, *C*_*c*_ = 1.0 kN·s/m) at *ε* = 1/2.

Modal properties of the first ten modes, obtained from the proposed analytical model and finite element simulation, are given in Table 3. The corresponding mode shapes are illustrated in Figure 4. Again, the two sets of results are found to agree well with each other. To assess the impact of cross-tie stiffness and damping on the modal behaviour of the studied symmetric DMT network, modal response of the same network but using rigid cross-tie is also listed in the same table. Noticing that the fundamental frequency of the target cable is 1.46 Hz, and the associated modal damping ratio is 0.5%, results in Table 3 show that when the target cable is connected to the neighbouring one using a rigid cross-tie, its fundamental frequency increases from 1.46 Hz to 1.68 Hz by 15% and the modal damping ratio from 0.5% to 0.61% by 22%. However, if a flexible damped cross-tie with a stiffness coefficient of *K*_*c*_ = 30.54 kN/m and damping coefficient of *C*_*c*_ = 1.0 kN·s/m is used instead, the fundamental frequency of the target cable would be increased by 6.2% to 1.55 Hz, whereas the modal damping ratio would be increased to 1.71% by 3.4 times. These suggest that using more rigid cross-tie would further enhance the in-plane stiffness of a cable network and thus the target cable, which agrees with the experimental observations by Yamaguchi and Nagahawatta [3] and Sun et al. [14]. Although the network fundamental mode is an in-phase global mode, the velocities at the cross-tie two ends are different due to the difference in the dynamic properties of the two main cables. Thus, unlike the twin-cable network case, damping existing in the cross-tie would offer nonzero damping force and help to dissipate more energy during oscillation.

In the case of the first out-of-phase global mode, the flexibility in the cross-tie reduces its modal frequency, so it is advanced from the third network mode (*f* = 3.375 Hz) in the rigid cross-tie case to the second network mode (*f* = 2.153 Hz) should a damped flexible cross-tie be used. Besides, since the relative velocity between the two cross-tie ends reaches its maxima in this oscillation mode, the large damping offered by the cross-tie leads to a drastic increment of its modal damping ratio from 0.34% in the rigid cross-tie case to 4.01% for a flexible damped cross-tie case.

The modal properties of the network local modes dominated by either the target cable or its neighbouring one are found to be independent of the cross-tie stiffness and damping (Modes 3, 4, 6, 9, and 10). In these cases, one of the main cables vibrates in an antisymmetric shape. Thus, the cross-tie happens to locate at the nodal point of the mode shape and would not have a role in altering modal properties. However, it is interesting to note that although the frequency of the 5th network mode, 4.42 Hz, is very close to the third modal frequency of the isolated target cable, which is 4.38 Hz (*Ω*_*o*_ = 3.0*π*), the modal damping ratio jumps by roughly 7 times from 0.16% (*ξ*_eq_ = *ξ*_1_/3) for a single cable to 1.11% when it is networked. As can be seen from Figure 4, when the network oscillates in this mode, the target cable vibrates in its 3rd mode and is dominant. The cross-tie is located at the maximum deformation location of the target cable whereas the neighbouring cable is almost at rest. Therefore, in terms of energy dissipation, the damped cross-tie acts like a dashpot damper installed at the mid-span of the target cable and “rigidly” supported by the neighbouring cable. Similar phenomenon is also observed in the 7th and 8th modes of the cable network, of which the motion is mainly dominated by one cable with the other cable almost at rest.

### 3.3. Case Study 3: Asymmetric DMT Cable Network

**Figure 5:**
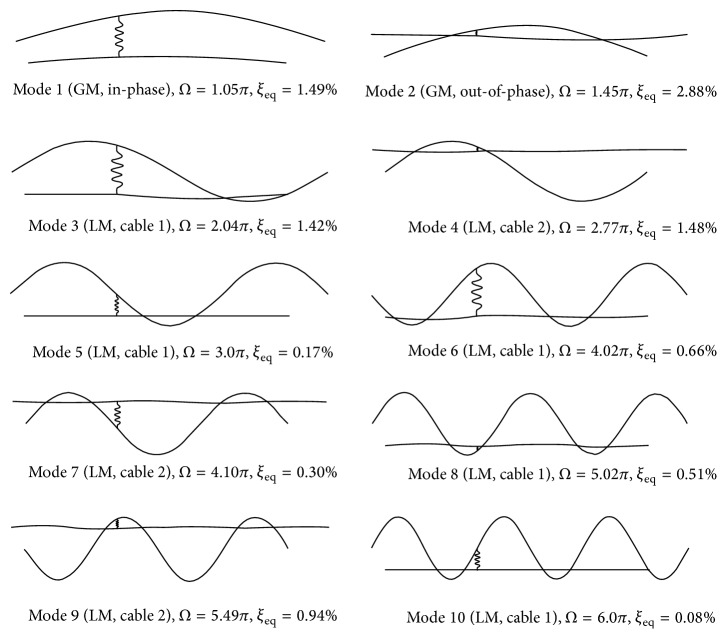
First ten modes of an asymmetric DMT two-cable network with a damped flexible cross-tie (*K*_*c*_ = 30.54 kN/m, *C*_*c*_ = 1.0 kN·s/m) at *ε* = 1/3.

The same two main cables in the symmetric DMT cable network of Section 3.2 are rearranged in this section such that the left and the right offsets of the neighbouring cable with respect to the target cable (Figure 1) are, respectively, 3 m and 9 m. In addition, the cross-tie is relocated to one-third span of the target cable from its left support; that is, *ε* = 1/3. These changes in the layout lead to an asymmetric DMT cable network.  Table 4 lists the modal properties of the first ten network modes obtained from the proposed analytical model and numerical simulation. A good agreement between the two sets can be clearly seen. In addition, the modal analysis results of a corresponding rigid cross-tie network are also given in the same table for the convenience of comparison. The mode shapes of these ten modes are depicted in Figure 5.

Results in Table 4 indicate that, by replacing the rigid cross-tie with a damped flexible one, the frequency of the network fundamental mode, which is an in-phase global mode, decreases whereas its modal damping ratio increases drastically. The target cable has a fundamental frequency of 1.46 Hz and a modal damping ratio of 0.50%. When it is connected with the neighbouring cable using a rigid cross-tie, the modal frequency is increased to 1.65 Hz by 13% and the damping ratio to 0.58% by 16%. However, if the cross-tie has properties of *K*_*c*_ = 30.54 kN/m and *C*_*c*_ = 1.0 kN·s/m, the increment of its fundamental frequency and damping ratio becomes 4.8% to 1.53 Hz and approximately three times to 1.49%, respectively. The same phenomenon can be observed in Mode 2, which is an out-of-phase global mode; that is, although using a damped flexible cross-tie would reduce the gain in network stiffness to some extent, it could greatly improve the energy dissipation capacity of the formed cable network. This is consistent with the existing experience (e.g., [3, 14]). In addition, it should be noted that, similar to the symmetric layout case in Section 3.2, such a change in cross-tie properties would lead to excitation of more local modes dominated by one of the main cables. This could be mainly attributed to the increased flexibility in cross-tie, which offers more freedom to one cable from the constraint of the other so it can oscillate more independently. Among the first ten modes listed in Table 4, the number of local modes increases from 2 to 8. Mode 4 and Mode 9 in the rigid cross-tie case are dominated, respectively, by the 3rd and the 6th mode of an isolated target cable. The position of cross-tie at *ε* = 1/3 happens to coincide with the nodal point of these single cable modes. Thus, the modal properties of these two local modes are not affected by the cross-tie stiffness and damping except that they become the 5th and the 10th modes when a damped flexible cross-tie is used instead.

**Figure 6:**
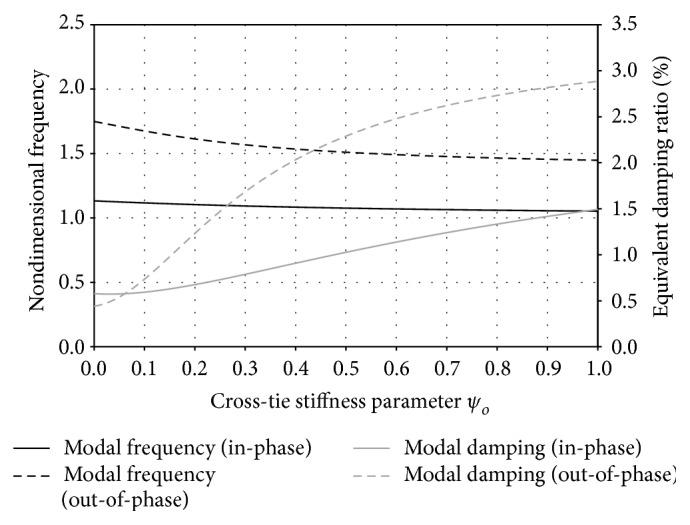
Effect of undamped cross-tie stiffness parameter *ψ*_*o*_ on modal frequency and modal damping ratio of the lowest in-phase and out-of-phase global modes of an asymmetric DMT cable network.

Besides, a parametric study is conducted for this asymmetric DMT cable network to better understand the effect of cross-tie stiffness and damping on the modal frequency and damping ratio of the network global modes.  Figure 6 depicts the modal property variation of the lowest network in-phase global mode and out-of-phase global mode with respect to the undamped cross-tie stiffness parameter *ψ*_*o*_. In the analysis, the cross-tie damping coefficient is assumed to be *C*_*c*_ = 1.0 kN·s/m, whereas *ψ*_*o*_ varies from 0 (rigid) to 1.0, which is a typical range of cross-tie stiffness on real cable-stayed bridges [7]. It can be seen from Figure 6 that overall the modal properties of the out-of-phase global mode are more sensitive to the cross-tie stiffness. As expected, the frequencies of both global modes decrease monotonically with the increase of cross-tie flexibility. Within the studied range of *ψ*_*o*_, the frequency of the in-phase global mode decreases by 7% while that of the out-of-phase global mode drops roughly by 18%. In terms of modal damping ratio, since the linear viscous damping model is used for describing cross-tie damping property, a more flexible cross-tie would result in higher relative motion velocity between the cross-tie two ends and thus more contribution to energy dissipation of the oscillating main cables in the network. It is also interesting to note from the figure that while the damping ratio increment rate of the in-phase global mode is more steady when *ψ*_*o*_ increases from 0 to 1, that of the out-of-phase global mode appears to be gradually decreasing as the cross-tie becomes more and more flexible. In general, the patterns of *ψ*_*o*_-Ω and *ψ*_*o*_-*ξ*_eq_ curves in Figure 6 imply that although using a more flexible cross-tie would cause some loss in network in-plane stiffness, the energy dissipation capacity could be greatly improved, which is beneficial for cable vibration control.

**Figure 7:**
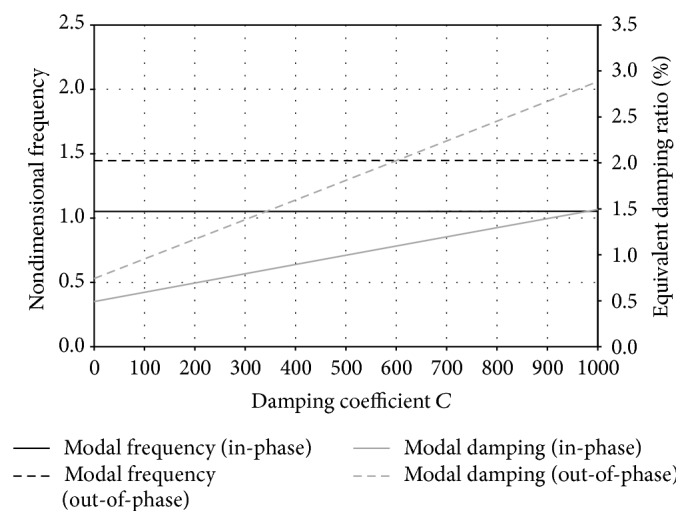
Effect of cross-tie damping coefficient *C* on modal frequency and modal damping ratio of the lowest in-phase and out-of-phase global modes of an asymmetric DMT cable network.

The influence of cross-tie damping level on the modal properties of the two lowest network global modes is shown in Figure 7. The modal frequencies of the two global modes are independent of the cross-tie damping level and remain as constants, whereas their modal damping ratios increase almost linearly with the increase of cross-tie damping. Again, the out-of-phase global mode is found to be more sensitive to change in cross-tie damping. By increasing *C*_*c*_ from 0 to 1.0 kN·s/m, the modal damping ratio of the lowest out-of-phase global mode increases from 0.44% to 2.88% by roughly 6.5 times, whereas that of the in-phase global mode increases almost three times from 0.58% to 1.49%.

## 4. Conclusions

Cross-tie solution has been successfully applied on site to suppress large amplitude bridge stay cable vibrations. Although it is understood that the dynamic behaviour of a cable network is highly dependent on the installation location, stiffness, and damping of cross-ties, only the effects of the former two on the network response have been investigated, whereas the impact of cross-tie damping has rarely been addressed in the past. To have a more comprehensive description of the network properties and better predict its dynamic response, in the current study, an analytical model of a cable network has been proposed by considering the cross-tie stiffness and damping, as well as the damping of the constituting main cables in the formulation. The impact of cross-tie stiffness and damping on cable networks having different configurations has been investigated by using the corresponding undamped rigid cross-tie networks as the reference base. The findings obtained from the current study are summarized as follows:

1. In the case of a twin-cable network, the modal properties of its in-phase global modes are independent of cross-tie installation location, stiffness, and damping. However, replacing rigid cross-tie with a damped flexible one could have a significant impact on the local LS and RS modes and render them to evolve into out-of-phase global modes. While the frequencies and damping ratios of local RS modes would all be altered by the cross-tie stiffness and damping, those associated with local LS modes depend on the cross-tie position. They would remain the same as those in the rigid cross-tie case if the cross-tie position coincides with the nodal point of a specific isolated main cable mode, but they would be altered if the cross-tie is installed at the off-nodal point location.
2. For more general two-cable networks having either symmetric or asymmetric layout, although, compared to rigid cross-tie case, the adoption of a damped flexible cross-tie would decrease the frequencies of network global modes, considerable increase of their modal damping ratio has been observed. Therefore, a careful balance between the loss in network in-plane stiffness and the gain in energy dissipation capacity should be achieved when selecting cross-tie stiffness and damping in the network design. In addition, such a change in the cross-tie properties is found to excite more local modes dominated by one of the main cables.
3. Results obtained from a parametric study indicate that the flexibility of a cross-tie would affect both frequency and damping of different cable network modes, whereas cross-tie damping would only affect the damping ratio of these modes. An approximate linear relation between the cross-tie damping and damping ratio of network global modes is observed.
4. Compared to network in-phase global modes, modal properties associated with out-of-phase global modes are more sensitive to the change of cross-tie stiffness and damping.

Overall, the findings of the current study imply that although using a more flexible cross-tie would cause some loss in the network in-plane stiffness, its effectiveness in improving the energy dissipation capacity could make it more beneficial in cable vibration control.

## Figures and Tables

**Table 1:** Comparison of modal properties of a twin-cable network with damped flexible or rigid cross-tie at *ε* = 1/3.

Mode	Damped flexible cross-tie					Rigid cross-tie		
	(*K*_*c*_ = 30.54 kN/m, *C*_*c*_ = 1.0 kN·s/m)					(*K*_*c*_ = ∞, *C*_*c*_ = 0)		
	*f* (Hz)		*ξ*_eq_ (%)		Mode shape	*f* (Hz)	*ξ*_eq_ (%)	Mode shape
	Analytical	FEA	Analytical	FEA				
1	1.46	1.46	0.50	0.50	GM, in-phase	1.46	0.50	GM, in-phase
2	1.63	1.63	3.34	3.35	GM, out-of-phase	2.18	0.33	LM, RS
3	2.91	2.91	0.25	0.25	GM, in-phase	2.91	0.25	GM, in-phase
4	3.02	3.02	2.49	2.49	GM, out-of-phase	4.37	0.17	LM, RS
5	4.37	4.37	0.17	0.17	GM, in-phase	4.37	0.17	GM, in-phase
6	4.37	4.37	0.17	0.17	GM, out-of-phase	4.37	0.17	LM, LS
7	5.83	5.82	0.13	0.13	GM, in-phase	5.83	0.13	GM, in-phase
8	5.88	5.87	1.19	1.19	GM, out-of-phase	6.56	0.11	LM, RS
9	7.28	7.27	0.10	0.10	GM, in-phase	7.28	0.10	GM, in-phase
10	7.33	7.31	1.03	1.03	GM, out-of-phase	8.74	0.08	LM, RS

GM: global mode, LM: local mode, LS: left segment mode, and RS: right segment mode.

**Table 2:** Comparison of modal properties of a twin-cable network with damped flexible or rigid cross-tie at *ε* = 2/5.

Mode	Damped flexible cross-tie					Rigid cross-tie		
	(*K*_*c*_ = 30.54 kN/m, *C*_*c*_ = 1.0 kN·s/m)					(*K*_*c*_ = *∞*, *C*_*c*_ = 0)		
	*f* (Hz)		*ξ*_eq_ (%)		Mode shape	*f* (Hz)	*ξ*_eq_ (%)	Mode shape
	Analytical	FEA	Analytical	FEA				
1	1.46	1.46	0.50	0.50	GM, in-phase	1.46	0.50	GM, in-phase
2	1.68	1.68	4.11	4.12	GM, out-of-phase	2.43	0.30	LM, RS
3	2.91	2.91	0.25	0.25	GM, in-phase	2.91	0.25	GM, in-phase
4	2.96	2.96	1.30	1.30	GM, out-of-phase	3.64	0.20	LM, LS
5	4.37	4.37	0.17	0.17	GM, in-phase	4.37	0.17	GM, in-phase
6	4.40	4.37	0.82	0.82	GM, out-of-phase	4.86	0.15	LM, RS
7	5.83	5.82	0.13	0.13	GM, in-phase	5.83	0.13	GM, in-phase
8	5.89	5.89	1.49	1.49	GM, out-of-phase	7.28	0.10	LM, RS
9	7.28	7.27	0.10	0.10	GM, in-phase	7.28	0.10	GM, in-phase
10	7.28	7.27	0.10	0.10	GM, out-of-phase	7.28	0.10	LM, LS

**Table 3:** Comparison of modal properties of a symmetric DMT two-cable network with damped flexible or rigid cross-tie at *ε* = 1/2.

Mode	Damped flexible cross-tie					Rigid cross-tie		
	(*K*_*c*_ = 30.54 kN/m, *C*_*c*_ = 1.0 kN·s/m)					(*K*_*c*_ = *∞*, *C*_*c*_ = 0)		
	*f* (Hz)		*ξ*_eq_ (%)		Mode shape	*f* (Hz)	*ξ*_eq_ (%)	Mode shape
	Ana.	FEA	Ana.	FEA				
1	1.55	1.55	1.71	1.72	GM, in-phase	1.68	0.61	GM, in-phase
2	2.15	2.15	4.01	4.01	GM, out-of-phase	2.91	0.25	LM, cable 1
3	2.91	2.91	0.25	0.25	LM, cable 1	3.37	0.34	GM, out-of-phase
4	3.98	3.98	0.40	0.40	LM, cable 2	3.98	0.40	LM, cable 2
5	4.42	4.41	1.11	1.12	LM, cable 1	5.04	0.21	GM, in-phase
6	5.83	5.82	0.12	0.12	LM, cable 1	5.83	0.13	LM, cable 1
7	6.03	6.02	1.29	1.29	LM, cable 2	6.74	0.17	GM, out-of-phase
8	7.31	7.30	0.72	0.72	LM, cable 1	7.97	0.20	LM, cable 2
9	7.97	7.96	0.20	0.20	LM, cable 2	8.41	0.13	GM, in-phase
10	8.74	8.72	0.08	0.08	LM, cable 1	8.74	0.08	LM, cable 1

**Table 4:** Comparison of modal properties of an asymmetric DMT two-cable network with damped flexible or rigid cross-tie at *ε* = 1/3.

Mode	Damped flexible cross-tie					Rigid cross-tie		
	(*K*_*c*_ = 30.54 kN/m, *C*_*c*_ = 1.0 kN·s/m)					(*K*_*c*_ = *∞*, *C*_*c*_ = 0)		
	*f* (Hz)		*ξ*_eq_ (%)		Mode shape	*f* (Hz)	*ξ*_eq_ (%)	Mode shape
	Ana.	FEA	Ana.	FEA				
1	1.53	1.53	1.49	1.49	GM, in-phase	1.65	0.58	GM, in-phase
2	2.11	2.11	2.88	2.89	GM, out-of-phase	2.55	0.44	GM, out-of-phase
3	2.97	2.97	1.42	1.42	LM, cable 1	3.49	0.35	GM, in-phase
4	4.04	4.03	1.48	1.48	LM, cable 2	4.37	0.17	LM, cable 1
5	4.37	4.37	0.17	0.17	LM, cable 1	5.00	0.23	GM, out-of-phase
6	5.85	5.85	0.66	0.66	LM, cable 1	5.97	0.26	GM, out-of-phase
7	5.98	5.97	0.30	0.30	LM, cable 2	6.43	0.14	GM, out-of-phase
8	7.30	7.29	0.51	0.51	LM, cable 1	7.54	0.14	GM, in-phase
9	8.00	7.99	0.94	0.94	LM, cable 2	8.74	0.08	LM, cable 1
10	8.74	8.72	0.08	0.08	LM, cable 1	9.05	0.14	GM, out-of-phase
